# Knowledge and Practice of Preventive Measures for Oral Health Care among Male Intermediate Schoolchildren in Abha, Saudi Arabia

**DOI:** 10.3390/ijerph17030703

**Published:** 2020-01-21

**Authors:** Saad Masood Al-Qahtani, Pervez Abdul Razak, Siraj DAA Khan

**Affiliations:** 1 Department of Preventive Dental Sciences, College of Dentistry, Najran University, Najran 11001, Saudi Arabia; sdkhan@nu.edu.sa; 2 Faculty of Dentistry, School of Health Sciences, University of Georgia, Tbilisi 0171, Georgia; drpervez@gmail.com

**Keywords:** oral health, schoolchildren, knowledge, practice

## Abstract

The aims of this study were to evaluate oral health knowledge and assess the practice of preventive measures for oral health care among intermediate schoolchildren in Abha, Saudi Arabia. Information about oral health was collected through a questionnaire containing closed-ended questions, which was distributed to children of six randomly selected intermediate schools. Most (82.3%) of the schoolchildren were aware that good oral health is important for general health. The priority for oral health information was given to dentists (31.6%), whereas teachers were given the least priority (19.1%). About half (53.5%) of the schoolchildren reported that sweets are the cause of dental caries, and 47.1% of them related pain with dental caries. More than half (58.8%) took sweets between meals. Most of them (69.6%) visited a dentist because of pain. Two-thirds (66.9%) of the children did not brush their teeth daily, and most (78%) did not use dental floss. A large number (62.7%) of the schoolchildren stated that rinsing with water after each meal is the best way to keep their gums healthy. For boys in intermediate schools, properly designed oral health educational programs should be implemented to improve their knowledge and behavior toward oral health.

## 1. Introduction

Oral health is a state of being free from chronic orofacial pain, oral cancer, oral infection, periodontal (gum) disease, tooth decay, tooth loss, and other diseases that limit an individual’s capacity in biting, chewing, smiling, and speaking, as well as psychosocial well-being [1]. Good oral health maintains general health [1,2]. Oral diseases such as dental caries, periodontal diseases, and tooth loss are becoming more prevalent in low- and middle-income countries. They are also a significant problem in high-income countries [1,3]. Their increasing incidences are due to the adoption of a Western lifestyle and changing living conditions. Furthermore, their prevalence rates have increased in the last few years [1,4]. Dental caries are the most prevalent oral health problem globally, affecting 60% to 90% of schoolchildren [1,3,5].

Oral health knowledge is an essential prerequisite for health-related practices [2,6]. Oral diseases including dental caries and periodontal diseases are obviously related to behavior, and their prevalence rate decreased as oral hygiene practice increased. In addition, reducing sugar consumption is strongly associated with a reduction in caries prevalence [6]. Good oral health practice consists of the continuous implementation of two sets of behavior: utilization of dental services (regular dental checkup, oral health promotion, and professionally applied preventive means) and self-care habits (good oral hygiene, restriction of sugar intake, and application of fluoride products) [7,8]. To prevent oral health problems, it is recommended that adults should brush and floss their teeth at least once a day and have a regular oral health checkup. [8,9]. The recommended frequency of toothbrushing is twice a day with fluoride toothpaste [7].

Health education is the transmission of knowledge and skills that are necessary for the improvement of life quality, as it is a widely accepted approach for oral disease prevention. In addition, the goal of planned health education programs is to not only bring about new behaviors but also maintain and reinforce healthy behaviors that will improve individual and community health [10]. To emphasize a positive attitude toward oral health, schools should include oral health education programs in the curriculum of schoolchildren [5]. Before designing an effective program for oral health promotion, it is important to consider the current status of oral health knowledge among children. It is also expected that oral health education is based on the grounds that it will enhance these children’s oral health knowledge by transforming it into appropriate preventive behaviors, consequently resulting in better oral health [11].

Many studies on oral health knowledge and the practice of preventive measures among intermediate schoolchildren have been conducted. In China, rural schoolchildren lacked knowledge about dental caries, gum diseases, and fluoride use [12]. In Spain, 61.1% of 12-year-old children showed at least one decayed, filled or missing tooth, and their knowledge about gingivitis was poor [13]. In the United Arab Emirates, it was observed that in schoolchildren, when knowledge increased, practice also increased, which showed the relationship between knowledge and practice [14]. In Saudi Arabia, 59% of students attending the Jenadriyah festival in Riyadh had heard about dentists and oral health from their families [15]. Among intermediate and high school children in Jeddah, Saudi Arabia, 24% of them never visited a dentist, and pain was found to be the main reason for visiting one [9]. Schoolchildren in Rijal Alma (a governate in Aseer Province, Saudi Arabia) had a low level of oral health practice [2].

Oral health knowledge and oral hygiene practice assessment are still limited in other provinces of Saudi Arabia, including Abha, an urban area in the southwestern region and the capital of Aseer Province. No studies related to this topic have been carried out. Hence, the purposes of this study were to determine oral health knowledge and assess the practice of preventive measures among male schoolchildren of intermediate schools in the city of Abha.

## 2. Materials and Methods

This study included 540, 12–16-year-old male schoolchildren from six randomly selected public and private intermediate schools that were geographically dispersed in every region of Abha city and used the stratified random sampling technique (see Supplementary 1).

The study was approved by the research ethical committee (SA2343-5A) of the Faculty of Dentistry, King Khalid University in Abha, Kingdom of Saudi Arabia. The list of schools was given by the General Administration of Education in Abha. The research proposal and questionnaire were sent to the General Administration of Education in Abha. Principals of the schools obtained written informed consent from the parents of all the schoolchildren.

All the respondents were requested to answer a self-administered questionnaire modified from that of Wyne et al. [11]. It was in Arabic language after being translated from English (see Questionnaire Supplementary 2). A pilot study was important to check the validity and comprehensibility of one questionnaire, which included 22 questions for 30 schoolchildren, and its results yielded an acceptable form with minor amendments based on comments from the schoolchildren. Five public schools and one private school were visited, and information about oral health knowledge was collected through a questionnaire that was distributed in the classrooms. It was emphasized to the participants that they should answer each question. After completion, the questionnaires were collected by one of the researchers who was present in the class in case any clarification was required. The questionnaire included 22 items in three sections. The first section was about demographical data, such as the age and nationality of the participants. The second section assessed oral health knowledge, and the third section evaluated the practice of oral health preventive measures.

The study data were analyzed using Statistical Package for the Social Sciences version 17. Descriptive statistical analysis was carried out. Chi-squared test was used to examine the association of results between two categorical variables, and a *p*-value of less than 0.05 was considered statistically significant. Results are depicted in the form of tables and charts.

## 3. Results

In this study, all 540 schoolchildren responded. Their ages ranged from 12 to 16 years (mean, 14; SD, 1.2) and all were male students. Most (86.2%) were Saudi children (Table 1).

**Figure 1:**
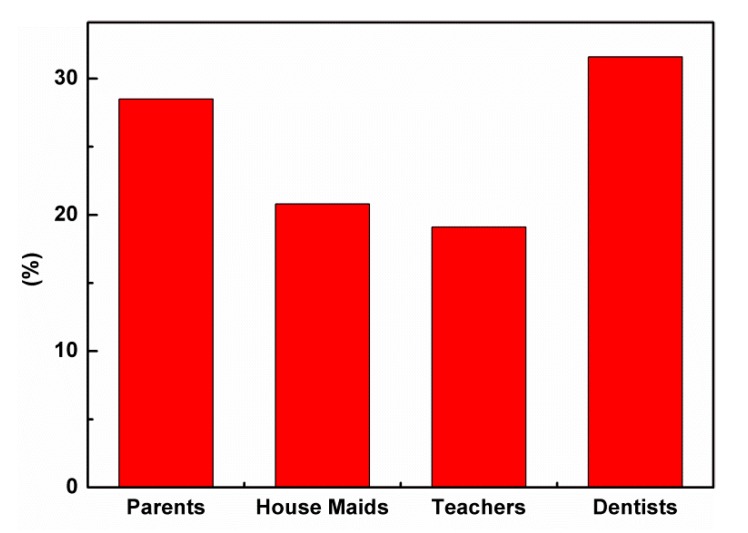
Source of oral health information.

**Figure 2:**
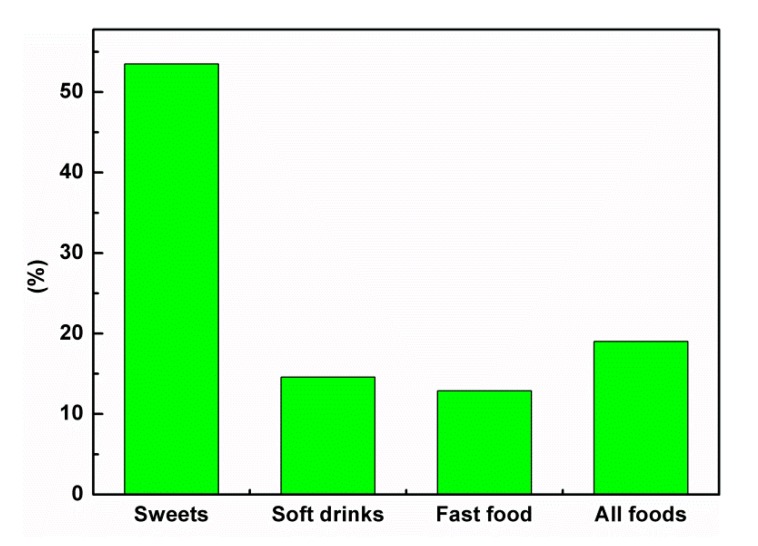
Causes of dental caries.

The schoolchildren’s responses about oral health knowledge are shown in Table 2. The vast majority (82.2%) of schoolchildren were aware that good oral health is important for general health. More than half (53.3%) of the schoolchildren said that the functions of the teeth are for chewing, speech, and appearance, and 18.5% of the children answered that the teeth are for chewing only. Dentists were the most popular (31.6%) source of oral health information, but some (28.5%) reported that their parents were the source of this information (Figure 1). About 63.4% of schoolchildren were not aware of fluoride, whereas 36.6% knew about it. Most (34.8%) who knew about it said that fluoride should be added to toothpastes for whitening the teeth. Approximately 53.5% of the schoolchildren realized that only sweets were the cause of dental caries, whereas 19% of them recognized that the cause of dental caries is not only sweets but also soft drinks, fast food, and fries (Figure 2). Less than half (47.1%) of the schoolchildren knew that one of their teeth was decayed when they experienced pain, and 80.7% knew that it is necessary to take care of their gums.

**Figure 3:**
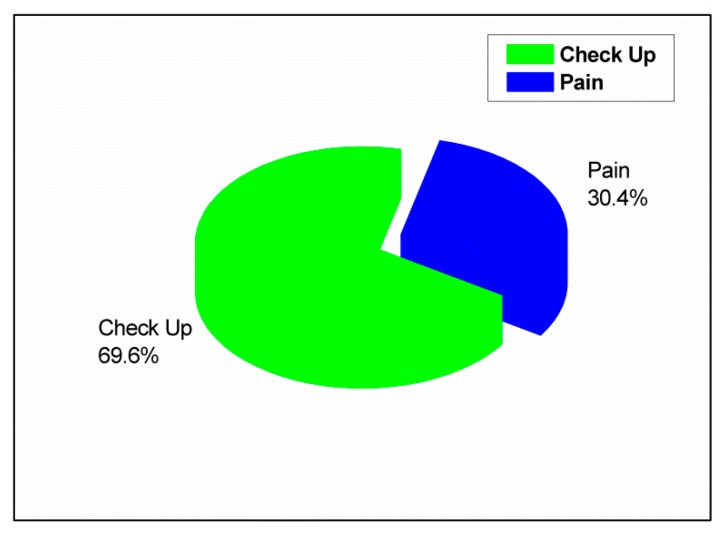
Reasons for visiting a dentist.

**Figure 4:**
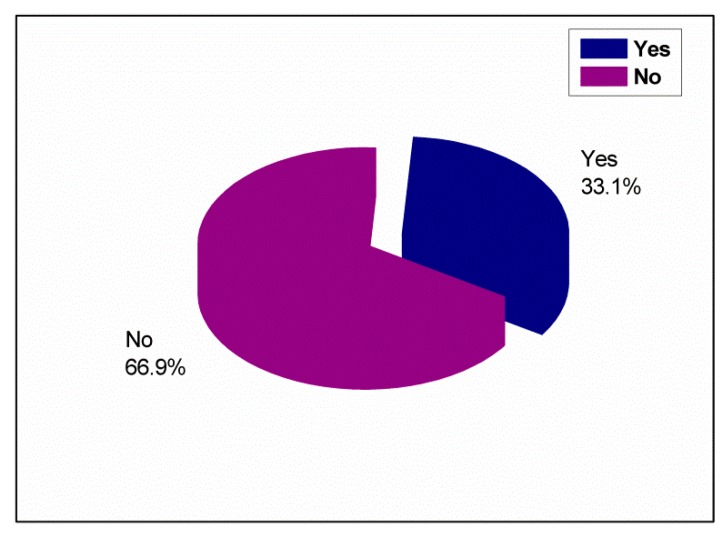
Toothbrushing.

The responses of the schoolchildren about the practice of oral health and preventive measures are summarized in Table 3. More than two-thirds (69.6%) of the schoolchildren visited a dentist when they experienced pain, as shown in Figure 3. Some (30.4%) visited a dentist for checkups; 45.5% visited a dentist occasionally, whereas 42.4% regularly visited a dentist once every six months. Less than half (48%) of the schoolchildren did not visit a dentist owing to carelessness, followed by fear among 24% of them. For cleaning their teeth, 79.4% of the schoolchildren used a toothbrush and toothpaste, whereas 17.8% used miswak (a piece of a branch or root of a tree that is used as a toothbrush). Most (66.9%) did not brush their teeth daily, as shown in Figure 4. However, 33.1% of the schoolchildren brushed their teeth daily; 50.2% brushed their teeth once daily, whereas 35.8% brushed their teeth twice daily. Among the schoolchildren who brushed their teeth in this study, 44.6% brushed their teeth at any time, whereas 43.1% brushed their teeth in the morning and evening. Among those who used a toothbrush, about 54.8% did not know which type they used. Only 30.1% of these schoolchildren used a soft brush. It was also found that only 22% of the schoolchildren used dental floss, and only 36.5% used mouthwash, whereas the rest did not. Different methods of keeping the gums healthy were practiced; 62.7% of the schoolchildren rinsed their mouth with water after meals, and 16.3% brushed their teeth.

Non-Saudi schoolchildren differed from Saudi students in the following ways: most (94.8%) of these schoolchildren visited a dentist regularly every six months, and 65.4% reported that the absence of dental problems was the reason for not visiting a dentist.

About 94.7% of the Non-Saudi schoolchildren cleaned their teeth with a toothbrush and toothpaste. Most (81.3%) brushed their teeth daily. Furthermore, 78.7% brushed their teeth twice daily and 82% brushed their teeth in the morning and evening. A soft dental brush was used by 72.1% of them. Most (52%) used dental floss, whereas nearly 73.3% used a mouthwash. Around 77.3% of these schoolchildren knew about fluoride, and 74.1% were aware of its role in preventing dental caries. Nearly 70.7% of these schoolchildren reported that sweets, soft drinks, fast food, and fries cause dental caries. Most (50.7%) ate sweets with meals. Most (72%) knew that one of their teeth was decayed when there were black or brown spots on the tooth. Nearly 69.3% replied that the best way to keep their gums healthy is by brushing their teeth. Parents were the most popular (54.6%) source of oral health information, followed by dentists (36%).

As shown in all the tables, relationships among the nationalities of the schoolchildren, oral health knowledge, and practice of preventive measures were observed. All these differences were found to be statistically significant (*p* < 0.001, *p* < 0.05).

## 4. Discussion

This study provided basic information about oral health knowledge and practice of preventive measures among male schoolchildren of intermediate schools in Abha. This information will help in designing oral health education programs for these schoolchildren to improve their knowledge and motivate them to practice the preventive measures. According to Zhu et al., preventive oral health care is better than a curative approach, as oral health education programs play the most important role in prevention, and schools are effective settings for this education [16]. Educational models are more effective, and nurses are important members of the team in schools’ oral health programs [17]. It is necessary that concerted efforts be made to implement preventive dental measures, thereby counteracting anticipated future dental problems [18]. A positive oral health attitude and behavior are closely related with good oral health [19]. Good oral health is important for getting a good job, improving self-esteem, and succeeding in life [20]. Several factors may affect the oral health behavior of an individual, including the acquisition of Western education, culture, and values [6,21]. Oral health knowledge of schoolchildren is influenced by socioeconomic factors, such as gender, ethnicity, type of school, income as well as the educational level of their parents [9,22,23]. A recent study by Calcagnile et al. indicated that not all parents in Italy are well informed with the oral hygiene of their children and, consequently, an oral health promotion is highly recommended [24]. Greater attention to visit the dentist regularly and maintain oral health is demonstrated by parents with a high educational level and sufficient income [22,23,25].

The vast majority of the schoolchildren had satisfactorily understood the importance of good oral health for the maintenance of general health, similar to the results of other studies [5,11,22,26,27,28,29,30,31]. Similar to reports by Al-Darwish et al. [22], information about the functions of the teeth, such as chewing, appearance, and talking, was reported by most of the schoolchildren, whereas chewing was considered as the most important function of the teeth in a study done in Riyadh, Saudi Arabia [11]. Appropriate knowledge about teeth functions will encourage schoolchildren to keep their teeth healthy. Dentists were the main source of dental information. A similar finding was reported by two studies [11,30], but it differed from two other studies [12,23], in which TV and radio as well as Internet were the main sources of oral health information. Studies carried out separately by Al Subait et al. [15], Ali et al. [3], and Al-Darwish et al. [22] described that family members, including parents, can be the source of this information. Parents must be educated in the right way in order to raise the oral health knowledge of their children. Teachers are the most popular source of dental health information, as students spend more time in school. Hence, reinforcing teachers’ knowledge for educating schoolchildren properly to improve their oral health information is highly recommended. Around two-thirds of the schoolchildren did not know about fluoride, whereas a few schoolchildren thought it was for whitening teeth. Many studies conducted worldwide found that most children have no idea about the roles of fluoride in strengthening the teeth and preventing dental caries [3,11,12,15,22,31]. Their results differ from those obtained by Amarlal et al. [5], Smyth et al. [13], and Graca et al. [30] who found that students knew about fluoride and that it could prevent dental caries and strengthen the teeth. Therefore, educating schoolchildren to enhance their knowledge about fluoride and its benefits is important for the prevention of caries. The knowledge about sweets as a cariogenic food was adequate, as reported by most of the schoolchildren. This finding is in agreement with many studies carried out in various populations [5,11,22,31]. Some schoolchildren knew that sweets, soft drinks, and fast food cause dental caries, as shown by certain studies [2,12,13,30], in which their subjects knew that sugary products cause dental caries. Thus, teaching schoolchildren about cariogenic foods and soft drinks is mandatory to minimize the consumption of sweets and other sugary products, especially sticky ones like candy and chocolates, in order to prevent tooth decay. Schoolchildren knew that one of their teeth was decayed when there was pain in it, as observed by most of the schoolchildren. These findings are in agreement with some studies [11,22] which showed that caries were detected in the later stages of their progress. Therefore, visiting a dentist regularly, such as once every six months for a checkup, is mandatory for schoolchildren to detect caries early and to adopt preventive treatment to stop the progress of caries. The schoolchildren’s knowledge about taking care of their gums to prevent gum diseases was satisfactory, as reported by several studies [11,22]; while other studies [5,31] stated that schoolchildren were unaware of the causes of gum disease. Hence, this knowledge will assist in reducing the incidence of periodontal diseases and tooth loss.

A large number of the schoolchildren ate sweets in-between meals, which is similar to a study by Zhu et al. [16], but dissimilar to a study by Ahmed et al. [29], who revealed that the preferred time for eating sweets is after meals. Hence, schoolchildren must reduce their consumption of sweets, especially between meals, as a method of preventing caries. Most of the schoolchildren who visited a dentist for checkup did so occasionally, in comparison with 19.1% of Indian schoolchildren who visited a dentist regularly every 6–12 months and 11.5% who visited occasionally [5]; 22% of Saudi schoolchildren in private schools visited a dentist regularly [9], 29% of 12-year-old Iraqi schoolchildren did so regularly [29], 25% of Saudi rural schoolchildren visited a dentist once every six months [2], and 35% of schoolchildren in Qatar visited a dentist every three months [22]. Most of the children visited a dentist during an emergency to obtain treatment when there was pain, which is in agreement with the findings of some previous studies [2,5,9,12,31]. This finding is in disagreement with a study that evaluated oral health awareness of Indian children in comparison to Western children, in which most of the Indian children visited a dentist because their parents had fixed an appointment with the dentist, whereas most Western children visited a dentist for follow-up treatment [27]; for instance, Swedish children visited a dentist for regular checkups [30]. Carelessness and fear were reported by most schoolchildren as the reasons for not visiting a dentist regularly; this was dissimilar to the findings of Amarlal et al. [5] and Al Subait et al. [15], who stated that “no pain felt” was a reason for not visiting a dentist. A study done by Farsi et al. [9] clarified that “no need” was the main reason for not visiting a dentist among male school students. Fear was the reason for not visiting a dentist, according to a study that was done in a rural Saudi area [2]. Pain was the driving factor for not visiting a dentist according to a study done by Blaggana et al. [31]. Therefore, these schoolchildren must be educated and motivated to visit a dentist once every six months for routine checkup, which is one of preventive measures for oral health care. This is due to the fact that the frequency of a check-up visit (once per six months) to a dentist for non-Saudi students (94.8%) within Abha, Saudi Arabia was less than that for Saudi students (14%), as shown in Figure Supplementary 1. Moreover, the frequency of a check-up visit (once per year) to a dentist for the whole sample in this study including Saudi and non-Saudi (6.7%) schoolchildren, was much less compared to other countries such as Qatar (35%) [22], Sweden (70.7%), Romania (46.8%), and Portugal (73%) (Figure Supplementary 2) [30]. A toothbrush with toothpaste was considered as the most important cleansing tool among a large percentage of the respondents, which was similar to the findings of numerous published studies [3,5,9,15,28,31]. Miswak was used by some schoolchildren, in comparison with 27%, 40%, and 32% of Saudi schoolchildren in Riyadh [28], Jeddah [9], and Aseer [2], respectively, and 23% of Pakistani schoolchildren [3]. Most of the schoolchildren did not brush their teeth daily, in agreement with a study done in China by Gao et al. [12], but the rest brushed their teeth, and most of them brushed their teeth once daily, as reported in several studies [2,3,12,22,28,31], yet other studies [5,16,27,30] found twice daily as the frequency of toothbrushing instead. A number of children thought that tooth brushing can be at any time, whereas some clarified that the teeth should be brushed in the morning and evening, which differ from the findings of several studies [3,5,16,31] that stated that morning time is desirable for toothbrushing. Most of the schoolchildren did not know the type of dental brush that they used, which is in disagreement with two studies done by Zhu et al. [16] and Blaggana et al. [31], who reported the use of a soft dental brush. Dental floss was not used by most of the schoolchildren, in agreement with many studies [2,5,9,28,31], but in disagreement with a study by Grewal and Kaur [27], who showed that more than half of American children flossed their teeth regularly. In addition, most of the schoolchildren were not mouthwash users, in agreement with some studies [5,9,31]. Thus, school dental health programs must educate schoolchildren about the proper method of brushing and brushing at least twice daily, that is, once in the morning when they wake up and once in the evening before going to bed, using a soft dental brush and fluoride toothpaste. Miswak is used by the Saudi population due to cultural and religious issues; therefore, schoolchildren should be taught the proper way of using it and should be advised to use dental floss and mouthwash as preventive measures along with brushing for good oral hygiene. Most of the schoolchildren reported that gum diseases could be prevented by rinsing the mouth with water after each meal. This finding is in conflict with other studies [2,5,16,22] conducted throughout the world, which reported toothbrushing as a preventive measure against gum diseases. Educating and motivating the schoolchildren to brush their teeth regularly are useful ways of removing plaque, which consequently results in healthy gums.

Compared to their Saudi counterparts, non-Saudi schoolchildren showed a clear difference in terms of oral health knowledge and practice of preventive measures in most of the answered items. All these differences were statistically significant owing to variations in eating and brushing habits as well as their cultures and the educational level of their parents. They had optimum oral health knowledge and practiced good preventive measures that enabled them to have better oral health. It should also be noted that in Saudi Arabia, health care, including dentistry, is provided freely without any cost for Saudi citizens. However, it should be noted that the findings of this study are not fully precise because the data collected in this study were self-reported by schoolchildren, which has a possibility of subjective bias from some respondents owing to misunderstanding of some items. In addition, the obligation to answer all questions and not having the “do not know” option can lead in the same way to unreliable results. This study could be improved in the near future by tackling the above-mention reasons, and carrying out an oral examination, which could be important for an objective assessment of the link between knowledge and the practice of oral health.

## 5. Conclusions

There is appropriate knowledge about some oral health topics but insufficient knowledge about others, and also poor practice of oral health preventive measures as seen in Saudi schoolchildren. Knowledge about fluoride and its role in the prevention of caries was limited. Knowledge about sweets as a reason for tooth decay was sufficient, but the knowledge of other foods that also cause dental caries, such as soft drinks and fast food, was insufficient. The practice of routine dental checkups was unsatisfactory. The practice of toothbrushing was not good, and its frequency (once daily) was still far behind the international recommendation (twice daily). Eating sweets between meals was reported as a bad habit, and the practice of preventive measures against gum diseases was not optimal. Non-Saudi schoolchildren had good dental health knowledge and positive oral hygiene behavior compared with the Saudi ones. The implementation of oral health education programs in schools and the practice of preventive measures are highly recommended in Abha to improve oral health knowledge. Further studies must be carried out in other parts of the Aseer Province.

## Figures and Tables

**Table 1:** Demographical data.

Items	No.	%	Mean	Standard Deviation
**Age groups**			14	1.2
12–13	16	5.3		
13–14	179	33.1		
14–15	101	18.7		
15–16	147	27.2		
≥16	97	15.5		
**Nationality**				
Saudi	465	86.2		
Non-Saudi	75	13.8		
**School type**				
Public	450	83.3		
Private	90	16.7		

**Table 2:** Oral health knowledge.

Question	Answer	All		Saudi		Non-Saudi		*p*-Value
		No.	%	No.	%	No.	%	
Do you know the importance of good oral health for general health?	Yes	443	82.2	372	80	71	94.6	*p* < 0.05
	No	97	17.8	93	20	4	5.4	
What are the functions of the teeth?	Chewing and eating	98	18.5	92	19.7	6	8	*p* < 0.001
	Speech	64	11.8	62	13.3	2	2.7	
	Appearance	88	16.2	82	17.7	6	8	
	All of the above	290	53.5	229	49.3	61	81.3	
Where did you get the information about dental health?	Parents	154	28.5	113	24.3	41	54.6	*p* < 0.001
	House maids	112	20.8	109	23.4	3	4	
	Teachers	103	19.1	99	21.3	4	5.3	
	Dentists	171	31.6	144	31	27	36	
Do you know the substance “fluoride”?	Yes	198	36.6	140	30.1	58	77.3	*p* < 0.001
	No	342	63.4	325	69.9	17	22.6	
Why should fluoride be added to toothpaste?	To give a pleasant taste	44	22.2	37	26.4	7	12.1	*p* < 0.001
	To whiten the teeth	69	34.8	63	45	6	10.3	
	To prevent dental caries	58	29.3	15	10.7	43	74.1	
	To act as a preservative	27	13.7	25	17.9	2	3.5	
Which of the following causes tooth decay?	Sweets	289	53.5	278	59.8	11	14.7	*p* < 0.001
	Soft drinks	79	14.6	72	15.5	7	9.3	
	Fast food	70	12.9	66	14.2	4	5.3	
	All of the above	102	19	49	10.5	53	70.7	
When do you know that your tooth is decayed?	Black and brown spots on the tooth	72	13.3	18	3.9	54	72	*p* < 0.001
	Cavity in the tooth	98	18.1	97	20.9	1	1.3	
	Pain in the tooth	254	47.1	243	52.2	11	14.7	
	Swelling around the tooth	116	21.5	107	23	9	12	
Do you know that it is necessary to take care of your gums?	Yes	436	80.7	367	78.9	69	92	*p* < 0.05
	No	104	19.3	98	21.1	6	8	

**Table 3:** Practice of preventive measures.

Items		All		Saudi		Non-Saudi		*p*-Value
Question	Answer	No.	%	No.	%	No	%	
When do you eat sweets?	With meals	50	9.3	12	2.6	38	50.7	*p* < 0.001
	In-between meals	318	58.8	309	66.4	9	12	
	Do not eat	33	6.1	14	3.1	19	25.3	
	After meals	139	25.8	130	27.9	9	12	
When do you visit a dentist?	Dental checkup	165	30.4	107	23.1	58	77.3	*p* < 0.001
	During an emergency when there is pain	375	69.6	358	76.9	17	22.7	
How often do you visit a dentist for checkup?	Once in six months	70	42.4	15	14	55	94.8	*p* < 0.001
	Once in a year	11	6.7	10	9.3	1	1.7	
	Once in two years	9	5.4	9	8.4	0	0	
	Occasionally	75	45.5	73	68.3	2	3.5	
Why do you not you visit a dentist?	Carelessness	257	47.6	227	48.9	13	17.3	*p* < 0.001
	Fear	130	24	114	24.5	9	12	
	No dental problems	121	22.4	95	20.4	49	65.4	
	Time-consuming	32	6	29	6.2	4	5.3	
How do you clean your teeth?	Toothbrush and toothpaste	429	79.4	358	77	71	94.7	*p* < 0.05
	Miswak	96	17.8	94	20.2	2	2.7	
	Toothpicks	9	1.7	8	1.7	1	1.3	
	Tooth powder and finger	6	1.1	5	1.1	1	1.3	
Do you brush your teeth daily?	Yes	179	33.1	118	25.4	61	81.3	*p* < 0.001
	No	361	66.9	347	74.6	14	18.7	
How many times do you brush your teeth daily?	Once	90	50.2	84	71.2	6	9.8	*p* < 0.001
	Twice	64	35.8	16	13.5	48	78.7	
	After each meal	20	11.2	14	11.9	6	9.8	
	More than three times	5	2.8	4	3.4	1	1.7	
When do you brush your teeth?	Morning only	12	6.7	7	5.9	5	8.2	*p* < 0.001
	Evening only	10	5.6	9	7.6	1	1.7	
	Morning and evening	77	43.1	27	22.9	50	82	
	At any time	80	44.6	75	63.6	5	8.1	
What type of toothbrush do you use?	Hard	15	8.4	14	11.9	1	1.7	*p* < 0.001
	Soft	54	30.1	10	8.5	44	72.1	
	Medium	12	6.7	2	1.6	10	16.4	
	Do not know	98	54.8	92	78	6	9.8	
Do you use dental floss?	Yes	120	22	81	17.4	39	52	*p* < 0.001
	No	420	78	384	82.6	36	48	
Do you use mouthwash?	Yes	197	36.5	142	30.5	55	73.3	*p* < 0.001
	No	343	63.5	323	69.5	20	26.6	
How do you keep your gums healthy?	Brushing teeth with mouth wash	88	16.3	36	7.7	52	69.3	*p* < 0.001
	Rinsing the mouth with water after meals	339	62.7	336	72.3	3	4	
	Using mouthwash only	54	10	50	10.8	4	5.3	
	Taking vitamins	59	11	43	9.2	16	21.4	

## Supplementary Materials

The following are available online at https://www.mdpi.com/1660-4601/17/3/703/s1, S1: Stratified Random Sampling Technique, S2: Questionnaire.
