# Supplementary material for: Knowledge and Practice of Preventive Measures for Oral Health Care among Male Intermediate Schoolchildren in Abha, Saudi Arabia
Source: Int J Environ Res Public Health. 2020 Jan 21;17(3):703. doi: 10.3390/ijerph17030703 (PMC7038016; doi:10.3390/ijerph17030703)
Supplement: Supplementary file 1 [file ijerph-17-00703-s001.pdf]

Supplementary File

Knowledge and Practice of Preventive Measures for Oral Health Care among Male Intermediate School Children in Abha, Saudi Arabia

Saad Masood Al-Qahtani [1*], Pervez Abdul Razak [2], Siraj DAA Khan [1]

[1] Department of Preventive Dental Sciences, College of Dentistry, Najran University, P.O. Box 1988, Najran 11001, Saudi Arabia; smqahtani@nu.edu.sa (S.A); sdkhan@nu.edu.sa (S.K)

[2] Department of Dentistry, School of Health Sciences, University of Gerorgia, Tbilisi, Gerorgia 0171; drpervez@gmail.com; (P.R)

* Correspondence: smqahtani@nu.edu.sa (S.A); Tel.: +96-650-455-2700

S1- Stratified Random Sampling Technique

We have used Sample size Calculator (https://www.calculator.net/sample-size-calculator.html) and this calculator computes the minimum number of necessary samples to meet the desired statistical constraints. Whereas Confidence Interval was 95% with 5% precision of error. Estimated Knowledge was 50% because We had no idea about it. Total Number of Schoolchildren was 10010, So the sample size was 370. We added 170 to ensure no problem might be occurred during study regarding adequacy of sample. We Selected 6 schools (5 public,1 Private) and targeted 90 student from each school.

.

S2- Questionnaire

Q1- Age…………………………….

Q2- Nationality …………………

Q3.Do you know the importance of good oral health for general health?

A. Yes
B. No

Q4. What are the functions of the teeth?

A. Chewing & eating
B. Speech
C. Appearance
D. All of the above

Q5.Where did you get the information about dental health?

A. Parents
B. House Maids.
C. Teachers.
D. Dentists

Q6.Do you know the substance "fluoride"?

A. Yes
B. No

Q7.Why should fluoride be added to toothpaste?

A. To give a pleasant taste.
B. To whiten the teeth.
C. To prevent dental caries.
D. To act as a preservative.

Q8.Which of the following causes tooth decay?

A. Sweets.
B. Soft drinks.
C. Fast food.
D. All of the above.

Q9.When do you know that your tooth is decayed?

A. Black & brown spots on the tooth.
B. Cavity in the tooth.
C. Pain in the tooth.
D. Swelling around the tooth.

Q10.Do you know that it is necessary to take care of your gums?

A. Yes
B. No

Q11.When do you eat sweets?

A .With meals
B. In between meals
C. Do not eat
D. After meals

Q12.When do you visit a dentist?

A. Dental checkup
B. During an emergency when there is pain

Q13.How often do you visit a dentist for checkup?

A. Once in six months
B. Once in a year
C. Once in two years
D. Occasionally

Q14.Why don't you visit a dentist?

A. Carelessness
B. Fear
C. No dental problems
D. Time-consuming

Q15.How do you clean your teeth?

A .Toothbrush and toothpaste
B . Miswak

C. Tooth picks
D. Tooth powder and finger

Q16.Do you brush your teeth daily?

A. Yes
B. No

Q17. How many times do you brush your teeth daily?

A. Once
B. Twice
C. After each meal
D. More than three times

Q18.When do you brush your teeth?

A. Morning only
B. Evening only
C. Morning and evening
D. At any time

Q19. What type of toothbrush do you use?

A. Hard
B. Soft
C. Medium
D. Don't know

Q20.Do you use dental floss?

A. Yes
B. No

Q21.Do you use mouthwash?

A. Yes
B. No

Q22.How do you keep your gums healthy?

A. Brushing teeth with mouth wash
B. Rinsing the mouth with water after meal

C .Using mouthwash only
D. Taking vitamins

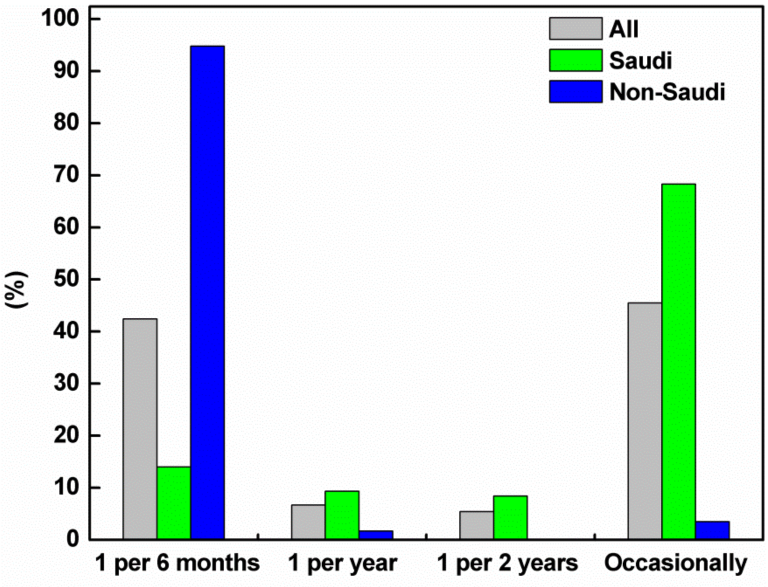

Figure S1: Frequency of visit to a dentist for Saudi and No-n Saudi students

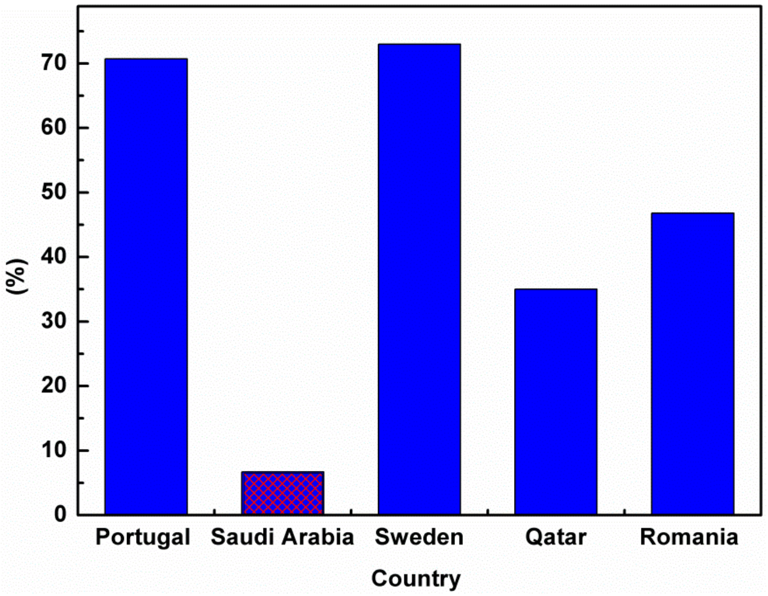

Figure S2: Frequency of visit to a dentist for students in Portugal, Saudi Arabia, Sweden, Qatar and Romania.
